# Respiratory pathogens in community acquired pneumonia

**DOI:** 10.6026/973206300210314

**Published:** 2025-03-31

**Authors:** Babita Kumari, Vijay Kumar

**Affiliations:** 1 Department of Microbiology, Patna Medical College, Patna, Bihar, India

**Keywords:** Respiratory pathogens, microbial detection, community acquired pneumonia (CAP)

## Abstract

Respiratory pathogens in community-acquired pneumonia are of interest. Hence, adults over 20 years of age who arrived at participating institutions' emergency rooms or outpatient clinics with pneumonia as clinical diagnosis were qualified for participation and evaluated for consideration in the study. Analysis shows that *K. pneumoniae*, *P. aeruginosa* and *S. pneumoniae* was the most common bacterial pathogens associated with community-acquired pneumonia. Most common viral pathogens associated with community-acquired pneumonia are influenza A and rhinovirus A.

## Background:

With a predicted prevalence of two to eleven episodes per 1000 persons in the industrialized world and a death rate of two percent to 14 percent, community-acquired pneumonia (community-acquired pneumonia) represents a widespread infectious illness [1, 2-3]. Given the variety of organisms that cause community-acquired pneumonia, after a microbiological identification has been established, wide-spectrum antimicrobial prophylaxis ought to be started in moderate infection or severe infections prior reducing to narrow range pathogen specific drugs [4, 5-6]. Tragically, hardly thirty percent to forty percent of individuals with community-acquired pneumonia may have a pathogen detected by current diagnostic techniques, making de-escalation rare in actuality. More rapid as well as accurate microbiological testing methods are needed for community-acquired pneumonia, especially for microorganisms and in the typical situation of antibiotics being administered before sampling, according to recent investigations [7, 8-9]. The choice of antibiotics for empirical use is based on information of the microorganisms that cause community-acquired pneumonia and this choice significantly affects the likelihood of a successful outcome [10, 11- 12]. Even with the advancement of better microbiological techniques in recent years, the root cause of community-acquired pneumonia remains poorly understood [13, 14, 15-16]. There aren't many published contemporary studies that have well-defined populations of patients, proper gathering of specimens before antibiotic therapy and comprehensive bacteriological as well as virological diagnoses. Although viruses are known to be significant contributors to community-acquired pneumonia in newborns and children [17, 18-19], it is unclear how these infections affect adults. The viral pathophysiology of community-acquired pneumonia may have previously been underappreciated due to a narrow spectrum of diagnostic approaches in the past, according to recent research centered on molecular testing [10-14]. Whether a viral infectious agent may result in pneumonia on its own or in combination with additional respiratory disease pathogens is still unknown. Certain respiratory viruses can infiltrate and multiply in the mucosal region of the lower respiratory system, according to previous research [15-17]. Furthermore, certain studies suggest that individuals with community-acquired pneumonia who receive treatment while being admitted to the hospital might be at a high risk of mixed infections [18, 19-20]. The isolation of new viruses, the recognition of pathogens which cannot be easily cultured and the recognition of pathogens during the course of the disease process have all been made possible by the arrival of molecular techniques with increased precision and sensitivity [20, 21, 22- 23]. Very few studies have reported the microorganisms that cause community-acquired pneumonia in India. Therefore, it is of interest to assess the respiratory pathogens in community-acquired pneumonia.

## Methods and Materials:

Adults over twenty years of age who arrived at participating institutions' emergency rooms or outpatient clinics with pneumonia as clinical diagnosis were qualified for participation and evaluated for consideration in the study.

## Criteria for diagnosis of pneumonia:

Fresh pulmonary infiltrates observed in thoracic imaging plus any or all of the following symptoms are indicative of pneumonia:

[1] A new or worsening coughing, with or without production of sputum and/or secretions from respiratory system with purulent nature

[2] Hypothermia or fever

[3] Indicators of systemic inflammation (elevated procalcitonin values or elevated C-reactive protein, leukocytosis more than 10,000 cells cm-3, leukopaenia less than 4000 cells cm-3 and bandemia more than ten percent)

## Participants were not included:

[1] If they have previously spent more than 48 hours in another inpatient facility (community hospitals, for example).

[2] Had pneumonia acquired in a hospital, which is pneumonia that appears 48 hours after being admitted to the hospital.

[3] Had been admitted to the emergency room within ninety days after suspecting tuberculosis or an infection with the human immunodeficiency virus.

[4] Were inhabitants of nursing homes?

[5] Couldn't give their consent

[6] Were either turned down to participate in this study or had already signed up for it within the preceding 30 days.

## Bacterial study:

After Gram staining, sputum specimen were deemed sufficient if they showed more than 25 leukocytes and less than ten epithelial cells every 100 x power microscopic field. Specimen sputum was taken for bacterial culture and handled using normal methods. Following the manufacturer's directions, 12 DNA was isolated out from two hundred µL of sputum specimen with a DNA isolation kit. The extracted DNA was kept at -20°C after being eluted in one hundred µL of elution buffer. By applying sequentially diluted DNA extracts, a standard curve was created to measure the quantity of bacterial DNA in every specimen. Each specimen's quantity of bacterial DNA was determined by directly extrapolating the Polymerase chain reaction cycle threshold (CT) readings to the total quantity of DNA. Detection of bacterial pathogens was carried out by sputum culture only, sputum Polymerase chain reaction only and both sputum culture as well as sputum PCR (polymerase chain reaction).

## Viral study:

Using standard viral culture methods, nasopharyngeal samples have been submitted for viral identification. MDCK, MRC-5 and MK2 cells received inoculation with cell suspension samples and the cells were then incubated for fourteen days at 35 degrees Celsius. Every two days, the cytopathic effect (CPE) of each culture tubes was examined. The Viral RNA kit was used for obtaining viral RNA from two hundred millilitres of respiratory samples and RT-Polymerase chain reaction kit was used to conduct reverse transcription procedures for complementary DNA creation. The viruses were detected using qPCR. Detection of viral pathogens was carried out by nasopharyngeal swab culture only, nasopharyngeal swab Polymerase chain reaction only and both nasopharyngeal culture as well as sputum PCR.

## Statistical analysis:

Group variations among categorical parameters were evaluated using either Fisher's exact test or chi-square test. One-way analysis of variance (ANOVA) was applied to continuous variables. The threshold for statistical significance was p < 0.05. Every probability was two-tailed. SPSS software (version 15.0, SPSS Inc., Chicago, IL, USA) was used for all statistical analyses.

## Results and Discussion:

440 patients with community-acquired pneumonia were evaluated. Different bacterial pathogens were identified in 269 (61.13%) patients. *K. pneumoniae* was the most common bacterial pathogen among community-acquired pneumonia patients being detected among 61 (14.2%) patients. Other common bacterial pathogens detected were *P. aeruginosa* being detected in 58 (13.6%) patients and *S. pneumoniae* being detected in 42 (9.9%) patients. Other bacterial pathogens were *H. influenzae* (6.2%), *S. aureus* (6.2%), *E. coli* (4.3%), *A. baumannii* (4.3%) and *M. catarrhalis* (3.4%). It was observed that maximum 40 cases out of 61 cases of community-acquired pneumonia being detected with *K. pneumoniae* were identified through sputum Polymerase chain reaction only, 8 cases identified through sputum culture only and 13 cases identified through both sputum culture as well as sputum PCR. Similarly, maximum cases of *P. aeruginosa* (28 out of 58), *S. pneumoniae* (36 out of 42), *E. col* (14 out of 18), *A. baumannii* (16 out of 18) and *M. catarrhalis* (10 out of 14) were identified through sputum Polymerase chain reaction only. However, most of the cases of *H. influenzae* (8 out of 26) and *S. aureus* (8 out of 26) were identified through sputum culture only. The findings were significant statistically (Table 1). In this study 440 patients with community-acquired pneumonia were evaluated. Different viral pathogens were identified in 165 (37.5%) patients. Influenza A was the most common viral pathogen among community-acquired pneumonia patients being detected among 53 (12.4%) patients. The common viral pathogens detected were Rhinovirus-A being detected in 31 (7.2%) patients and Influenza B and Rhinovirus-C being detected in 17 (3.9%) patients each. Other significant viral pathogens were HCoV (2.9%). It was observed that maximum 33 cases out of 53 cases of community-acquired pneumonia being detected with Influenza A virus were identified through both Polymerase chain reaction as well as culture, 20 cases identified through nasopharyngeal swab Polymerase chain reaction only. However, most of the cases of Influenza B (17 out of 17), HCoV (13 out of 13), Rhinovirus a (26 out of 31) and other viral pathogens were identified through nasopharyngeal swab Polymerase chain reaction only. The findings were significant statistically (Table 2).

Due to the vast range of organisms that cause community-acquired pneumonia , wide-spectrum antibiotic prophylaxis should be initiated in moderate or severe infections after a microbiological identification has been made, before switching to narrow-range pathogen-specific medications [12-15]. De-escalation is actually uncommon because, regrettably, only 30 to 40 percent of community-acquired pneumonia patients may have a pathogen identified by current diagnostic methods. According to recent studies, more reliable and quick microbiological testing techniques are required for community-acquired pneumonia, particularly for bacteria and in the common scenario of antibiotics being given prior to sampling [16-17]. Assessing respiratory pathogens and their microbiological identification in community-acquired pneumonia was the goal of this investigation. In this study 440 patients with community-acquired pneumonia were evaluated. Different bacterial pathogens were identified in 269 (61.13%) patients. *K. pneumoniae* was the most common bacterial pathogen among community-acquired pneumonia patients being detected among 61 (14.2%) patients. Other common bacterial pathogens detected were P.aeruginosa being detected in 58 (13.6%) patients and *S. pneumoniae* being detected in 42 (9.9%) patients. It was also observed that maximum cases of community-acquired pneumonia being detected with *K. pneumoniae* were identified through sputum Polymerase chain reaction only. Similarly, maximum cases of *P. aeruginosa*, *S. pneumoniae*, *E. col*, *A. baumannii*, *M. catarrhalis* were identified through sputum Polymerase chain reaction only. The findings of present study are having similarity with the findings of other studies [22, 23- 24]. These studies also found that *K. pneumoniae*, *P. aeruginosa*, *S. pneumoniae* being the most common bacterial pathogens associated with community-acquired pneumonia. Like our study, other studies also found that bacterial pathogens can be isolated by sputum culture and sputum Polymerase chain reaction [25, 26, 27- 28]. Based on knowledge of the microorganisms that cause community-acquired pneumonia, the selection of antibiotics for empirical usage has a substantial impact on the chances of a successful outcome. The underlying cause of community-acquired pneumonia is still not well known, despite recent improvements in microbiological approaches [10, 11, 12-13]. Few recent studies have been published with clearly defined patient groups, appropriate specimen collection prior to antibiotic treatment and thorough bacteriological and virological diagnosis [14, 15, 16, 17-18]. In this study different viral pathogens were identified in 165 (37.5%) community-acquired pneumonia patients. Influenza A was the most common viral pathogen among community-acquired pneumonia patients being detected among 53 (12.4%) patients. Other common viral pathogens detected were Rhinovirus-A being detected in 31 (7.2%) patients and Influenza B and Rhinovirus-C being detected in 17 (3.9%) patients each. It was observed that maximum cases of community-acquired pneumonia being detected with Influenza a virus were identified through both Polymerase chain reaction as well as culture. However, most of the cases of Influenza B, HCoV, Rhinovirus A and other viral pathogens were identified through nasopharyngeal swab Polymerase chain reaction only. The findings were significant statistically.

The findings of present study are having similarity with the findings of other studies [26, 27-28]. These studies also found that most common viral pathogens associated with community-acquired pneumonia are Influenza A and Rhinovirus-A. Other studies like our study found that Detection of viral pathogens was carried out by nasopharyngeal swab culture only, nasopharyngeal swab Polymerase chain reaction only and both nasopharyngeal culture as well as sputum Polymerase chain reaction [21-25]. It is unknown how viruses impact adults, despite the fact that they are known to have a major role in community-acquired pneumonia in infants and children [14-16]. According to new studies focused on molecular testing, the viral pathophysiology of community-acquired pneumonia may have previously been underestimated because of a limited range of diagnostic techniques [11-13]. It is still unclear whether a viral infectious agent alone or in conjunction with other respiratory disease pathogens can cause pneumonia. Previous studies have shown that some respiratory viruses can enter and grow in the mucosal area of the lower respiratory system [21-23]. Additionally, some research indicates that patients with community-acquired pneumonia who receive treatment during hospitalization may be particularly vulnerable to mixed infections [24, 25-26]. The advent of molecular techniques with greater sensitivity and precision has enabled the identification of pathogens during the course of the disease process, the isolation of novel viruses and the identification of diseases that are difficult to cultivate [27, 28].

## Conclusion:

Analysis shows that *K. pneumoniae*, *P. aeruginosa* and *S. pneumoniae* was the most common bacterial pathogens associated with community-acquired pneumonia. Most common viral pathogens associated with community-acquired pneumonia are influenza A and rhinovirus-A.

## Supplementary material:

No

## Author contribution:

Each author has made a substantial contribution to the conception or design of the work, acquisition, analysis and interpretation of data and has drafted the work and substantively revised it.

## Funding:

This research received no external funding.

## Figures and Tables

**Table 1:** Detection of bacterial pathogen in patients being diagnosed with community-acquired pneumonia

	***K. pneumoniae***	***P. aeruginosa***	***S. pneumoniae***	***H. influenzae***	***S. aureus***	***E. coli***	***A. baumannii***	***M. catarrhalis***	***M. pneumoniae***	**Others**	**Total**
No (%) of patients Having positive detection (n=440)	61 (14.2)	58 (13.6)	42 (9.9)	26 (6.2)	26 (6.2)	18 (4.3)	18 (4.3)	14 (3.4)	4 (0.91)	02 (7.6)	269 (61.13)
Sputum culture only	8	6	4	16	10	-	-	2	0	26	
Sputum Polymerase chain reaction only	40	28	36	8	8	14	16	10	4	0	
Both culture and PCR	13	24	2	2	8	4	2	4	-	6	

**Table 2:** Detection of viral pathogen in patients being diagnosed with community-acquired pneumonia

	**Influenza A**	**Influenza B**	**Parainfluenza**	**HCoV**	**Rhinovirus-A**	**Rhinovirus-B**	**Rhinovirus-C**	**Adenovirus**	**Respiratory syncytial virus**	**Human metapneumovirus**	**HSV-1**	**Total**
No (%) of patients having positive detection (n=440)	53 (12.4)	17 (3.9)	4 (0.91)	13 (2.9)	31 (7.2)	5 (0.94)	17 (3.9)	5 (0.94)	9 (2.1)	9 (2.14)	2 (0.5)	165 (37.5)
Nasopharyngeal swab culture only	-	-	-	-	-	-	-	-	-	-	2	
Nasopharyngeal swab Polymerase chain reaction only	20	17	2	13	26	5	17	3	5	9	0	
Both Polymerase chain reaction and culture	33	0	2	0	5	0	0	2	4	0	0	
